# Noninvasive Continuous Glucose Monitoring Using Multimodal Near-Infrared, Temperature, and Pressure Signals on the Earlobe

**DOI:** 10.3390/bios15070406

**Published:** 2025-06-24

**Authors:** Jongdeog Kim, Bong Kyu Kim, Mi-Ryong Park, Hyoyoung Cho, Chul Huh

**Affiliations:** 1 Digital Biomedical Research Division, Electronics and Telecommunications Research Institute (ETRI), Daejeon 34129, Republic of Korea; bongkim@etri.re.kr (B.K.K.); deardol@etri.re.kr (H.C.); chuh@etri.re.kr (C.H.); 2 Terrestrial and Non-Terrestrial Integrated Telecommunications Research Laboratory, Electronics and Telecommunications Research Institute (ETRI), Daejeon 34129, Republic of Korea; mrpark@etri.re.kr

**Keywords:** noninvasive, continuous glucose monitoring, multimodal signals, earlobe, near-infrared spectroscopy, diffused transmission, wearable sensor

## Abstract

This study investigates a noninvasive continuous glucose monitoring (NI-CGM) system optimized for earlobe application, leveraging the site’s anatomical advantages—absence of bone, muscle, and thick skin—for enhanced optical transmission. The system integrates multimodal sensing, combining near-infrared (NIR) diffuse transmission with temperature and pressure sensors. A novel Multi-Wavelength Slope Efficiency Near-Infrared Spectroscopy (MW-SE-NIRS) method is introduced, enhancing noise robustness through the slope efficiency-based parameterization of NIR signal dynamics. By employing three NIR wavelengths with distinct scattering and absorption properties, the method improves glucose detection reliability, addressing tissue heterogeneity and physiological noise in noninvasive monitoring. To validate the feasibility, a pilot clinical trial enrolled five participants with normal or pre-diabetic glucose profiles. Continuous glucose data capturing pre- and postprandial variations were analyzed using a 1D convolutional neural network (Conv1D). For three subjects under stable physiological conditions, the model achieved 97.0% Clarke error grid (CEG) A-Zone accuracy and a mean absolute relative difference (MARD) of 5.2%. Across all participants, results showed 90.9% CEG A-Zone accuracy and a MARD of 8.4%, with performance variations linked to individual factors such as earlobe thickness variability and physical activity. These outcomes demonstrate the potential of the MW-SE-NIRS system for noninvasive glucose monitoring and highlight the importance of future work on personalized modeling, sensor optimization, and larger-scale clinical validation.

## 1. Introduction

Glucose is the primary energy source for the human body, and when it is deficient, it can cause brain damage or death. Chronic hyperglycemia can lead to various complications, such as diabetic ketoacidosis, neuropathy, and retinopathy [1,2]. As conventional methods for blood glucose management, devices such as self-monitoring of blood glucose (SMBG) and minimally invasive continuous glucose monitoring (MI-CGM) systems are commonly used. SMBG measures blood glucose at a single point in time using a small blood sample obtained from the fingertip. In contrast, MI-CGM continuously monitors glucose levels over a certain period using a small sensor with a thin needle that is inserted subcutaneously and attached to the skin. While MI-CGM offers convenience, it is limited by tissue damage, infection risk, pain, and ongoing replacement costs [3].

Noninvasive blood glucose measurement technologies are classified as either single/intermittent or continuous monitoring [4,5]. This work focuses on continuous glucose monitoring (CGM), encompassing both minimally invasive (MI) and noninvasive (NI) methods. In clinical practice, MI-CGM systems (e.g., Dexcom G7) are typically referred to as simply “CGM,” while NI-CGM refers to technologies that automatically measure and record blood glucose at regular intervals without skin penetration.

Despite their potential, noninvasive blood glucose measurement methods have struggled to achieve clinical accuracy due to interference from skin, tissue, and other physiological factors. However, recent advances in multimodal sensor fusion [6,7] and nonlinear AI algorithms [8,9,10,11] have significantly improved the performance. For example, GlucoTrack, which measures ultrasonic, electromagnetic, and thermal properties of the earlobe, has shown promising clinical results [6]. Additionally, a review by Chan et al. [12] found that most studies since 2005 have used optical technologies, with random forests and artificial neural networks being the most common algorithms.

Most noninvasive glucose measurement studies have reported clinical results using devices applied to the finger or hand [10,11,13,14,15,16,17,18,19], despite these areas being suboptimal for continuous monitoring due to their high mobility. Proper evaluation of NI-CGM technology requires wearable sensors placed on body sites suitable for long-term use, yet clinical studies on near-infrared (NIR)-based NI-CGM systems for stable regions such as the wrist, upper arm, or earlobe are limited. For example, Geng et al. [7] tested a multisensor wrist/arm device combining impedance spectroscopy, NIR sensors, and environmental monitoring, while Valero et al. [20] evaluated the GlucoCheck device, which uses a 650 nm red laser through a finger or earlobe clip, in eight participants.

The visible and NIR wavelength ranges have been widely studied for glucose monitoring, with diffuse-reflectance spectroscopy [10,21,22,23] commonly used due to the limited feasibility of direct transmittance at most body sites. NIR spectroscopy detects glucose via overtone and combination bands of functional groups such as O-H (hydroxyl group), C-H (methyl/methylene group), and C=O (carbonyl group). Olesberg et al. [24] compared glucose detection in the combination band region (2000–2500 nm) and the first overtone region (1538–1818 nm). While Olesberg focused on these spectral regions, Ghazaryan et al. [25] adopted optoacoustic spectrometry at 1250 and 1650 nm, which measures laser-induced ultrasonic waves, and Fathimal et al. [26] explored 940/1050 nm while proposing 1300–1390 nm as a promising range for reducing scattering in anatomically complex tissues. However, noninvasive glucose detection in such heterogeneous tissues as fingers and wrists remains challenging due to interference from hemoglobin, water, and tissue heterogeneity. This underscores the advantage of alternative sites, such as the earlobe, which, owing to its thin stratum corneum, uniform structure, and dense capillary network [27], has demonstrated reduced scattering artifacts and improved suitability for optical glucose sensing [28].

In this study, we present a multimodal NI-CGM sensor system that measures NIR signals, temperature, and pressure at the earlobe. Using diffused transmission at three wavelengths (1310/1370/1625 nm), the system detects blood glucose-specific signals and collects data every 30 s. Key contributions include the following:

1. The MW-SE-NIRS Algorithm: This algorithm combines slope efficiency-based parameterization and post-warmup normalization to reduce hardware-induced variations and signal-to-noise variability.
2. Multi-Channel Fusion: Multi-channel fusion integrates three wavelength channels optimized for earlobe tissue properties, reducing scattering artifacts through cross-channel compensation.
3. The Low-Complexity Conv1D Model: The model achieves reliable glucose prediction with limited training data, leveraging streamlined architecture for biomedical applications.
4. Clinical Feasibility: The clinical feasibility was validated on five healthy subjects (400–700 frames/subject over 4–6 h), highlighting the potential for optimization despite challenges in individual/environmental variability.

This paper is organized as follows: Section 2 details the methodologies, including the sensor design, earlobe optical properties, MW-SE-NIRS algorithm, data preprocessing (normalization, outlier removal), and neural network architecture. Section 3 analyzes the frame data characteristics and Conv1D performance, as well as future directions to address generalization challenges. Section 4 concludes with the technical contributions.

## 2. Methodology and Design

### 2.1. System Overview

**Figure 1:**
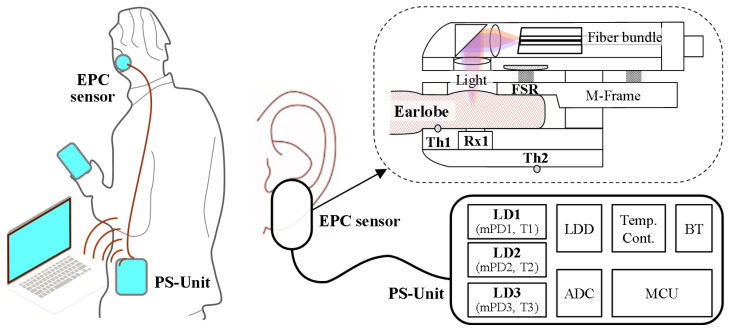
System architecture of the EPC sensor, comprising NIR, skin temperature, and skin pressure sensors. The PS-Unit is designed as a wearable device for the waist.

The NI-CGM sensor system in this study consists of an earlobe-parallel clip (EPC) sensor worn on the earlobe and a portable sensor unit (PS-Unit) worn on the waist, as shown in Figure 1, with the two devices connected via optical and electrical cables. The EPC sensor integrates several components, including an optical receiver (Rx1) for measuring diffused-transmission signals through the earlobe, a force-sensitive resistor (FSR) for detecting compression pressure on the earlobe, and two thermistors: Th1 for measuring the earlobe skin temperature and Th2 for monitoring the case and ambient temperatures. Additionally, the EPC incorporates a fiber-optic bundle to deliver multi-wavelength near-infrared signals to the tissue. This configuration enables the synchronized acquisition of optical, thermal, and mechanical data essential for robust glucose monitoring.

The EPC sensor is designed to accommodate varying adult earlobe thicknesses through an M-Frame and spring mechanism that leverages elastic force. This innovative design ensures uniform, gentle pressure distribution across the earlobe. A key feature of the EPC sensor is its ability to continuously monitor the spring pressure via the parallel ear-clip structure and integrated FSR sensor, ensuring consistent performance. Due to this structural design, the sensor simultaneously acquires multiple signals in real time: forward NIRS signals correlated with blood glucose levels, pressure, and temperature. During initial wear, FSR-derived pressure readings reflect individual earlobe thickness variations, while subsequent fluctuations captured during monitoring arise from movement artifacts.

The PS-Unit performs multiple functions: generating pulsed signals at three wavelengths (λ_1_, λ_2_, λ_3_) using laser diode (LD) sources (LD1–LD3), acquiring signals from multimodal sensors (Rx1, FSR, Th1, Th2), regulating the light source temperature, processing data via a microcontroller unit (MCU), and enabling Bluetooth communication to a computer or smartphone. The fiber-optic bundle connecting the EPC sensor and PS-Unit incorporates 8° angled end facets to minimize back-reflections and stabilize the LD output. Within the EPC sensor, the 3-channel optical system directs light onto the earlobe, where tissue absorption and scattering attenuate most photons. A fraction undergoes forward-direction multiple scattering, detected as diffuse-transmission signals by Rx1.

Each LD source in the PS-Unit was integrated with a monitoring photodiode (mPD) and a temperature sensor mounted on a thermoelectric cooler (TEC) within a packaged module. The Rx1 in the EPC sensor was integrated with a temperature sensor (T4) on a TEC in a separate packaged module. During continuous measurements under preset operating conditions, the three mPDs (mPD1–mPD3) independently track the output power of their corresponding LDs (LD1–LD3), while the Rx1 measures the diffuse-transmitted intensity through the earlobe. Simultaneously, the four temperature sensors (T1–T4) monitor the temperatures of LD1–LD3 and the Rx1, respectively.

Prior to developing the NI-CGM sensor system, we performed a systematic analysis of optical responses to glucose concentration variations. Using aqueous and solid phantom models [29], we identified the optimal NIR wavelengths for glucose detection. These foundational studies informed the selection of three laser diodes (LD1–LD3) in the PS-Unit, emitting λ_1_ = 1310 nm, λ_2_ = 1370 nm, and λ_3_ = 1625 nm. NIR spectral analysis (900–1700 nm) of the glucose solutions revealed that λ_2_ exhibited the highest sensitivity to the concentration changes, while λ_1_ and λ_3_ demonstrated comparable sensitivity levels.

### 2.2. Optical Properties of Earlobe and Wavelengths

**Figure 2:**
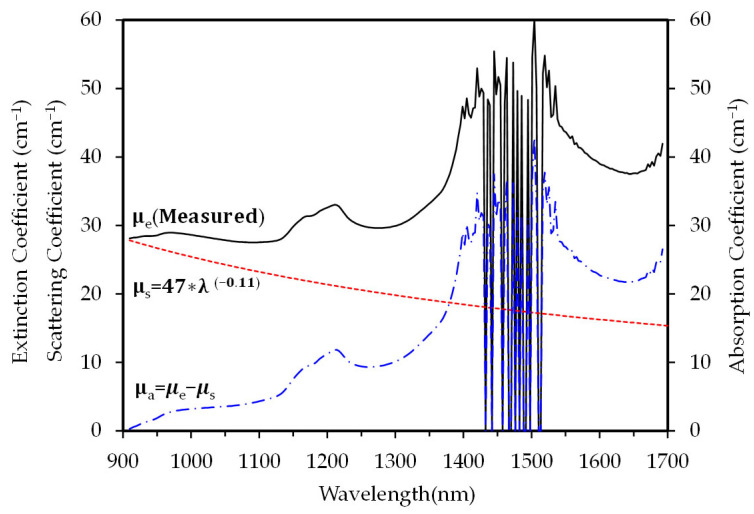
Comparison of estimated scattering and absorption coefficients with extinction coefficients derived from measured transmission spectra in the human earlobe.

Earlobe morphology (shape, size, thickness) varies significantly across individuals due to their pliable tissue structure. To ensure measurement consistency, we employed a digital thickness gauge with a spring-loaded circular disk matching the EPC sensor’s contact area, achieving 0.01 mm resolution. The earlobe thickness was measured in triplicate in 16 adult participants (age range: 20–60 years; 11 males, 5 females), yielding a mean thickness of 3.91 mm (range: 2.7–4.8 mm). Prior to implementing NI-CGM, we characterized the optical properties of human earlobes by measuring near-infrared transmission spectra using an experimental setup consistent with prior studies [29]. Figure 2 compares the extinction, scattering, and absorption coefficients derived from transmission spectra across the 900–1700 nm spectral range for a participant with a 4.8 mm thick earlobe. In the 1400–1550 nm subrange (excluded from the analysis due to dominant water absorption artifacts), the observed noise peak arises from short spectrometer integration times optimized for other spectral regions.

Light transmission in biological tissues is attenuated by absorption and scattering from cellular and molecular components. Transmittance decreases exponentially with the extinction coefficient (μₑ), defined as the sum of the absorption (μₐ) and scattering (μₛ) coefficients multiplied by the tissue thickness (t): μ_e_ = (1/t) ln(T/c), where T is the measured transmittance. The compensation parameter (c), critical for correcting instrumental losses and thickness measurement inaccuracies, can be normalized to c = 1 for relative extinction coefficient comparisons.

Optical scattering in biological tissues primarily arises from two mechanisms: Rayleigh and Mie scattering. Rayleigh scattering dominates when the particle dimensions are smaller than the incident wavelength, while Mie scattering occurs with larger particles. For instance, red blood cells—the smallest human cells—typically measure 7–8 μm in diameter, comparable to near-infrared wavelengths, and thus exhibit Mie scattering. In contrast, most other tissue components (e.g., adipocytes, fibroblasts) span tens of micrometers, further contributing to wavelength-dependent scattering behavior.

In the NIR region, the scattering coefficient (μ_s_) follows a power-law decay function: μ_s_ = Kλ^−h^, as demonstrated by Filatova et al. [30]. For this study, we adopted the parameters K = 47 and h = 0.11, with the primary focus on light attenuation at 900 nm (Figure 2). The absorption coefficient (μ_a_) is derived from the relationship μ_a_ = μ_e_ − μ_s_, where μ_e_ represents the extinction coefficient. An analysis of the earlobe transmittance revealed wavelength-dependent scattering and absorption behaviors, with μ_s_/μ_a_ ratios of 1.8, 1.1, and 0.7 at λ_1_ (1310 nm), λ_2_ (1370 nm), and λ_3_ (1625 nm), respectively. By leveraging these three wavelengths—each exhibiting distinct μ_s_/μ_a_ ratios—we developed an integrated model to predict blood glucose levels through diffuse-transmission signal analysis.

**Figure 3:**
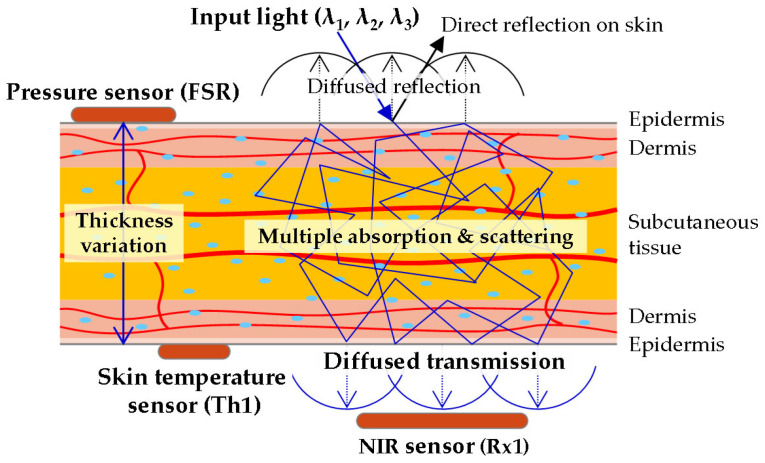
Illustrations of optical behavior through the components of the earlobe.

The NIR spectrum (1300–1650 nm) is particularly advantageous for probing biological tissues due to its reduced scattering and absorption coefficients, enabling deeper optical penetration. Consequently, the earlobe—with an average thickness of 3.91 mm (range: 2.7–4.8 mm)—exhibits sufficient transmittance at these wavelengths. Figure 3 illustrates the layered structure of the earlobe (epidermis, dermis, subcutaneous tissue) and the optical interactions of incident NIR light. Diffuse transmission effectively traverses the earlobe, allowing for the assessment of the interstitial fluid and capillary blood glucose levels within subcutaneous and dermal tissues. In contrast, diffuse reflection is dominated by strong surface signals from the epidermis and dermis, masking weaker contributions from deeper subcutaneous layers. This mechanistic rationale underscores the superior accuracy of diffuse-transmission-based NI-CGM, as it minimizes surface interference while capturing glucose-correlated signals from deeper vascularized tissues.

### 2.3. MW-SE-NIRS Algorithm

#### 2.3.1. Signal Parameterization

Conventional NIRS measurements predominantly rely on the received light intensity and transmittance/reflectance data from biological tissues. In contrast, slope efficiency (SE) measurement offers enhanced noise robustness, providing superior reliability. This study employed the SE parameter—defined as the slope of the linear region in the LD current–output curve—to analyze the tissue-induced variations in the linear response of the received optical signals.

**Figure 4:**
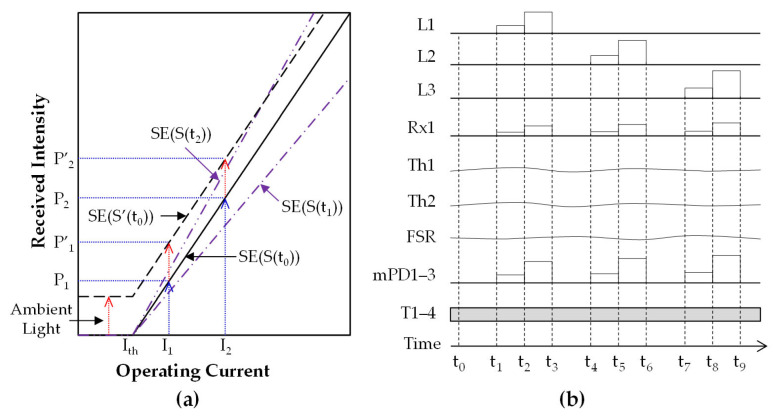
Signals in the MW-SE-NIRS algorithm: (**a**) concept of slope efficiency using received intensity over the earlobe in a single-wavelength channel; (**b**) signal processing of raw frame data from multiple sensors.

As illustrated in Figure 4a, the received optical intensities at driving currents I_1_ and I_2_ are denoted as P_1_ and P_2_, respectively. The SE is defined as follows:

SE = ΔP/ΔI = (P_2_ − P_1_)/(I_2_ − I_1_). (1)

The LD output exhibits linear behavior within a specific current range above the threshold current (Ith), a characteristic preserved even when light propagates through biological tissue. Experimental validation confirmed that earlobe-transmitted signals maintain linearity under short-duration, low-power LD operation (several milliwatts), provided that the tissue properties remain stable during measurements. However, prolonged monitoring may introduce nonlinearities due to dynamic physiological changes, such as blood glucose fluctuations.

The solid line represents the reception intensity as a function of LD bias in the biological tissue state (S(t)) at an arbitrary time (t), with the slope efficiency denoted as SE(S(t)). The long-dashed line corresponds to state S′(t_0_), where external noise light is added to S(t_0_), as indicated by the red-dashed arrow; in this case, the slope remains unchanged, so SE′(t_0_) = SE(t_0_). If changes in the external light during a single SE measurement can be disregarded, one of the main advantages of the SE is its resilience to ambient light. This demonstrates that ambient light does not affect the SE value, making the method highly effective for practical applications. Consequently, accurate measurements can be consistently obtained regardless of variations in lighting due to weather changes or different illumination levels.

Here, SE(S(t_1_)) and SE(S(t_2_)) represent the slope efficiency values corresponding to two distinct tissue states at times t_1_ and t_2_, respectively. Changes in biological tissue states—such as blood glucose levels and body temperature—alter the light extinction characteristics over time due to dynamic scattering and absorption effects. These changes modulate the extinction coefficient in the biological tissue (as explained in Section 2.2) and result in variations in the SE values. These findings advance the development of NI-CGM by demonstrating the utility of a slope efficiency-based NIRS approach, which enhances robustness against environmental interference.

#### 2.3.2. Signal Processing for Multimodal Sensors in MW-SE-NIRS

In practical applications, the slope efficiency—reflecting the extinction coefficient—can be directly calculated using Equation (1) by measuring the received power levels through biological tissue under two distinct input power conditions. The sensor system utilizes a three-channel LD light source with wavelengths λ_1_, λ_2_, and λ_3_, generating signals designated as L1 = LD(λ_1_), L2 = LD(λ_2_), and L3 = LD(λ_3_). As shown in Figure 4b, step pulses from these light sources (L1–L3) are sequentially generated and synchronized across time intervals (t_0_–t_9_), enabling the simultaneous measurement of SE signals and multimodal sensor data at the receiver. Each wavelength channel produces two consecutive step pulses—low-current (I1) and high-current (I2) phases—with adjustable pulse widths and inter-channel intervals. The receiver (Rx1) captures optical signals, while monitor photodiodes (mPD1–mPD3) track the output intensities from the LD1–LD3 modules. Temperature monitoring encompasses the LD module temperatures (T1–T3), receiver temperature (T4), earlobe skin temperature (Th1), and sensor case temperature (Th2). Concurrently, an FSR continuously monitors the earlobe compression pressure. The mechanistic insights elucidate the signal processing framework of MW-SE-NIRS, a core algorithm enabling a robust performance in our sensor system. This synchronized acquisition of optical, thermal, and mechanical parameters ensures robust signal correlation for accurate glucose prediction.

The PS-Unit is meticulously designed to generate NI-CGM data at 30 s intervals using the MW-SE-NIRS algorithm and transmit them via Bluetooth to a laptop computer. This systematic design achieves an hourly acquisition rate of 120 frames. In the MW-SE-NIRS method, the SE value for each wavelength channel is derived by averaging 700 measurements sampled at 500 Hz during the step-pulse width. Concurrently, the step-pulse protocol systematically measures the reception intensities from mPD1–mPD3 and the Rx1 using 16-bit analog-to-digital converters (ADCs). For each step-pulse phase, the SE per channel is calculated as follows: SE(λ, t) = ΔP(λ, t)/ΔI(λ, t), where ΔP(λ, t) and ΔI(λ, t) denote the differences in the received intensity and driving current between consecutive steps, respectively.

For the three wavelengths (λ_1_, λ_2_, λ_3_), the time-dependent SE values are expressed as follows: SE1(t) = SE(λ_1_, t), SE2(t) = SE(λ_2_, t), SE3(t) = SE(λ_3_, t). Since the generated output power from LD1–LD3 is separately observed by the mPDs, the system efficiency (SE) characteristics from mPD1 to mPD3 are labeled as m-SE1(t) to m-SE3(t), indicating the stability of the input power before entering the earlobe. The diffuse-transmitted power through the earlobe is also separately measured by the Rx1, and the SE characteristics of the Rx1 are labeled as dt-SE1(t) to dt-SE3(t), corresponding to LD1–LD3 under time-dependent operation (Figure 4b).

The SE signal strength depends on the driving conditions of the light source, the optical properties of the biological tissue, and the amplification gain of the receiver. When the driving conditions and amplification gain remain constant, SE variations primarily reflect changes in tissue properties. However, practical sensor systems exhibit channel-specific differences in driving conditions and gain. To eliminate the dependency on initial settings, the normalized SE signal—rather than the raw SE—is utilized, which inherently cancels systematic offsets, enabling robust comparisons across time and subjects.

The normalized slope efficiency (NSE) is defined as the ratio of the SE at an arbitrary time (t) to its value at a reference time (t_0_), corresponding to the 20th frame, as detailed in Section 3.1. For each wavelength channel (λ_1_, λ_2_, λ_3_), the m-NSE signals from the mPD1–mPD3 and dt-NSE signals from the Rx1 are calculated using Equations (2) and (3):

m-NSE#(t) = m-SE#(t)/m-SE#(t_0_), # = 1, 2, 3 for λ_1_, λ_2_, λ_3_. (2)

dt-NSE#(t) = dt-SE#(t)/dt-SE#(t_0_), # = 1, 2, 3 for λ_1_, λ_2_, λ_3_. (3)

The m-NSE signals primarily monitor the stability of the generated power in each LD channel rather than detecting glucose levels. Conversely, the dt-NSE signals show significant correlation with blood glucose concentrations, and each channel (dt-NSE1 to dt-NSE3) can be utilized for glucose prediction. However, as described in Section 2.5, the performance of the dt-NSE signals varies randomly across individual channels and subjects. Through a comprehensive comparative analysis, we established that the diffuse-transmission integral approach (dt-IA123)—which integrates three-wavelength dt-NSE signals (dt-NSE1, dt-NSE2, dt-NSE3) into a single variable—provides optimal accuracy for blood glucose prediction. The dt-IA123 is defined as follows:

dt-IA123(t) = ln[dt-NSE1(t) × dt-NSE2(t) × dt-NSE3(t)] = ln[dt-NSE1(t)] + ln[dt-NSE2(t)] + ln[dt-NSE3(t)].(4)

Equation (4) effectively integrates features from multi-channel NIR reflectance spectroscopy while reducing multicollinearity. Furthermore, logarithmic transformation is applied to improve the regression model accuracy by stabilizing the variance and enhancing the data normality.

### 2.4. Clinical Trial and Data Collection

The clinical trial was approved by the ETRI Ethics Committee and conducted in accordance with all relevant guidelines and regulations. Each volunteer wore an EPC sensor on their left earlobe and carried a PS-Unit pouch at their waist. Reference blood glucose data for model training and evaluation were collected using a Dexcom G7 CGM sensor, which was worn on the subjects’ upper arms. Hourly fingerstick samples were obtained with an SMBG device (HANDOK, BAROZEN II) to calibrate the G7 CGM sensor during fasting and to compare it with the NI-CGM under carefully maintained steady-state conditions.

The Dexcom G7 CGM sensor provides improved performance, with an accuracy of an 8.7% MARD compared to earlier models, and automatically records blood glucose data every 5 min for convenient data collection. However, initial use may result in inaccurate readings due to potential tissue damage. To ensure stable blood glucose measurements, participants attached the sensor to their upper arms at least 24 h prior to the NI-CGM measurement. As the NI-CGM and CGM sensors have different measurement intervals, time synchronization is required for accurate comparison. We applied linear interpolation to convert CGM data from 5 min intervals to 30 s intervals, ensuring a more precise match than simply assigning a single CGM value to 10 frames of NI-CGM data over a 5 min period.

In this study, NI-CGM data were collected from five volunteers: three with normal glucose levels and two classified as pre-diabetic, with HbA1c readings between 5.7% and 6.4%. Table 1 summarizes the relevant information for the five volunteers, including earlobe thicknesses and NI-CGM data. Each volunteer is identified as V1 through V5. The earlobe thickness for each subject was measured both before and after wearing the EPC sensor during the clinical test. The percentage thickness variation for individual subjects was calculated using the following formula: Thickness Variation (%) = 100 × (Thickness_after_ − Thickness_before_)/Thickness_after_. Interestingly, we observed that the earlobe thickness varied among the subjects, with changes ranging from 0.74% to −8.72% under the fixed force applied by the EPC sensor during continuous wear. These characteristics are related to the prediction accuracy of blood glucose, which will be discussed in Section 3. The earlobe thickness was measured three times for each event using the same digital gauge described in Section 2.2, and the average values are reported in Table 1.

This study utilized two NI-CGM sensor systems: the EPC sensor and PS-Unit #1 were applied to three subjects (V1–V3), while the EPC sensor and PS-Unit #2 were used for two subjects (V4–V5). Both systems shared the same MW-SP-NIRS algorithm but were assembled only with minor variations in the operational parameters, including the LD current, optical coupling loss, and receiver amplifier gain. This design strategy was implemented to verify the reproducibility of the NI-CGM sensor platform, with the goal of applying multiple NI-CGM sensor configurations to larger populations in future studies.

The clinical trial for each subject was conducted continuously over several hours from morning to afternoon, encompassing lunchtime. Participants were permitted to move freely within the laboratory while wearing the NI-CGM sensor to simulate real-life conditions. However, most chose to remain seated, engaging in activities such as using personal laptops, conversing, resting, or eating. All participants received identical carbohydrate-rich meals during the measurement period, consuming 70–100% of the provided food within approximately 30–40 min per meal.

Continuous measurements over 4–6 h per subject yielded 2522 frames of earlobe data—capturing three-wavelength NIRS, temperature, and pressure profiles—alongside 252 glucose readings from five participants. Each frame integrates the NI-CGM parameters (glucose levels, temperature, pressure) with synchronized sensor system status data (operating temperature, current consumption, and signal output intensity).

### 2.5. Feature Engineering and Neural Network Design

#### 2.5.1. Feature Selection and Exploratory Analysis

The NI-CGM sensor system provides normalized data (NT1–4, NTh1–2, NFSR, m-NSE1–3, and dt-NSE1–3 data) and generates dt-IA123 data through signal processing. These data are categorized into three groups for the machine learning model training and sensor stability verification:

- The kernel group: This group includes dt-NSE1–3 and dt-IA123 data, which show strong correlations with glucose levels.
- The assistant group: This group comprises NTh1 and NFSR data to monitor skin temperature and earlobe pressure, which may interfere with kernel group data.
- The additional group: This group contains NT1–4, m-NSE1–3 data to assess the sensor system stability, along with NTh2 to track ambient temperature changes during testing.

The parameters in the kernel and assistant groups represent the primary features contributing to nonlinear behavior and mutual interference effects. The parameters in the additional group can also interfere with those in the kernel and assistant groups if the system operates unreliably during measurement.

In preliminary studies, we evaluated the blood glucose prediction performance using NI-CGM data with low-complexity neural networks, specifically multilayer perceptron (MLP) and 1D convolutional (Conv1D) models [31,32]. Additionally, channel-specific NSE datasets were analyzed using multiple linear regression (MLR)—a linear model with multiple independent variables—to compare its performance against nonlinear approaches. While MLR offers advantages such as rapid training and interpretability through explicit coefficient analysis, its inherent linearity limits accuracy when modeling nonlinear relationships between independent variables (e.g., dt-NSE1–3, dt-IN123 signals) and dependent variables (blood glucose levels). As no significant prediction results were observed with these linear models, they are excluded from the results discussed in the subsequent sections.

In contrast, MLP and Conv1D models excel at capturing complex nonlinear interactions. MLP leverages activation functions (ReLU, sigmoid, tanh) to model nonlinear relationships, independently learning features from multivariate data or variable interactions. However, MLP struggles with temporal dependencies and is prone to overfitting, particularly with high-dimensional inputs or suboptimal hidden-layer configurations. Conv1D, adapted for time-series analysis, efficiently processes multidimensional data by learning localized temporal patterns through 1D convolutional filters.

#### 2.5.2. Design of Conv1D Models and Hyperparameter Optimization

NIR signals interact with biological tissues through complex mechanisms, necessitating nonlinear machine learning models for accurate blood glucose prediction. Common algorithms for noninvasive glucose monitoring include random forests and neural networks [10]. While both Conv1D and random forests employ nonlinear structures to learn patterns, their approaches differ fundamentally: Conv1D automatically extracts spatiotemporal features through convolutional operations, whereas random forests mitigate overfitting via ensemble decision trees.

Based on prior comparative analyses, we selected the Conv1D architecture from the CNN series for this study, as it effectively captures temporal dependencies in NI-CGM data while demonstrating superior robustness against overfitting. This approach aligns with recent advancements in biosignal processing, where modified ResNet34 architectures using Conv1D layers demonstrate enhanced performances in photoplethysmography (PPG)-based glucose monitoring by preserving temporal dynamics [33].

To conduct a fundamental performance analysis using the pilot clinical trial data, we optimized the learning process with state-of-the-art deep learning techniques. The loss function gradients were computed via backpropagation, enabling iterative model parameter updates, and the mean-squared error (MSE) was employed as the loss function to minimize the squared difference between the predicted and actual glucose values. Parameter optimization utilized the Adaptive Moment Estimation (Adam) optimizer, which adaptively estimates first-order (mean) and second-order (variance) moments to adjust per-parameter learning rates, thereby achieving rapid convergence and stable training. The batch size was set to 16 to balance the memory efficiency and gradient stability, and the model was trained for 100 epochs to ensure proper convergence, with the learning rate fixed at 0.001 to optimize the gradient steps. After training, the model was converted to the Open Neural Network Exchange (ONNX) format to enhance the interoperability across deep learning frameworks and hardware platforms. The ONNX conversion process allows for further optimization for specific environments using dedicated ONNX tools.

The Conv1D model is a type of convolutional neural network specifically designed for one-dimensional input data and relies on key hyperparameters, such as input channels, output channels, and kernel size. The model architecture consists of two Conv1D layers that transform up to 4 input channels into 32 output channels, followed by an average pooling layer to produce the final output. In optimization experiments, we determined that a kernel size of 3, a stride of 1, and padding of 1 were effective for maintaining the output size while managing boundary effects.

This structure allows for flexibility in adjusting the model’s depth through the configuration of the input and output channels. By utilizing learned kernel weights during inference, the design improves both the speed of convergence during training and the accuracy of predictions. The inclusion of the average pooling layer also reduces the spatial dimension of the feature maps, helping to prevent overfitting and enhance computational efficiency. Given that the Conv1D model is designed for relatively low-complexity and small-scale datasets, we set the number of convolutional layers to two (num_conv_layers = 2) as a hyperparameter and selected a kernel size of three (kernel_size = 3).

It is crucial to verify the minimum amount of training data required for each neural network model to effectively optimize the hyperparameters. For the Conv1D model, the minimum training data is generally calculated as B × L_min_ × F, where B is the batch size, L_min_ is the minimum sequence length, and F is the number of features. With a batch size of B = 16 and a minimum sequence length of L_min_ = 5 (determined through the optimization of the kernel size, stride, and batch size, as explained above), the Conv1D model requires a minimum of 240 to 320 data points for F = 3 to 4 features, respectively.

We constructed a dataset of the key features influencing blood glucose prediction by training and testing multiple machine learning models, using individual data categorized into three variable groups (as described in Section 2.5.1). Our analysis revealed that employing a single integrated parameter (dt-IA123) within the kernel group—rather than treating dt-NSE1–3 as three separate variables—yielded more stable results, as justified by Equation (4). Concurrently, parameters NT1–4 and m-NSE1–3 from the additional group were recursively eliminated due to their non-correlation with blood glucose levels and their propensity to increase prediction errors. These findings align with the sensor system’s design framework, which maintains strict control over the light source output and operating temperatures under fixed conditions, as detailed in the stability analysis within the Results and Discussion Section. Furthermore, overlapping temperature monitoring parameters (NTh1 and NTh2) did not improve the performance of the tested machine learning models. Consequently, while these additional-group parameters were excluded from the current analysis, they hold potential for improving the efficacy of advanced learning architectures (e.g., temporal convolution networks or attention-based transformer networks) in future studies. By integrating features from the kernel group and the auxiliary dataset, this study established a consolidated feature set using the integral approach outlined in Equation (4). With F = 3 optimized features, the Conv1D model requires a minimum of 240 data points for training, ensuring robust hyperparameter optimization, as per the B × L_min_ × F framework.

#### 2.5.3. Pearson Correlation and Multicollinearity

**Figure 5:**
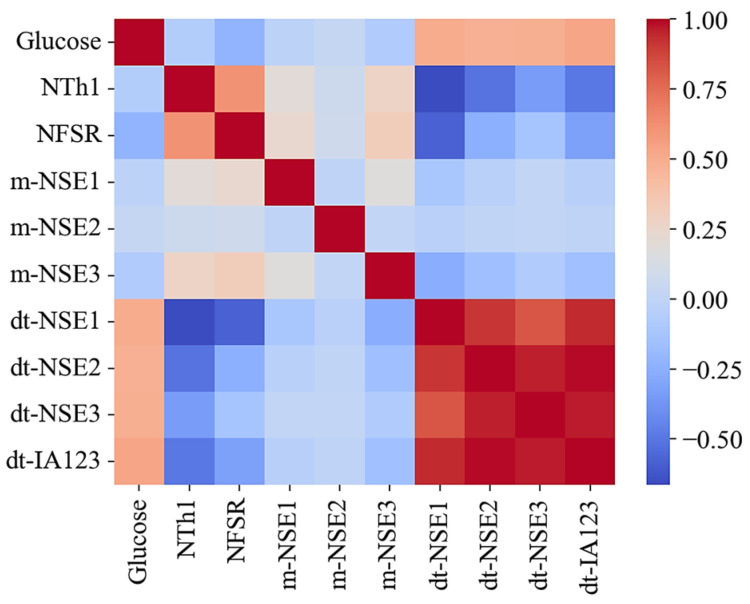
Correlation matrix showing relationships among variables in the dataset of subject V1.

Figure 5 illustrates the Pearson correlation coefficients among the key variables in the frame dataset of subject V1. For comparison with dt-IN123, m-NSE1–3 and dt-NSE1–3 are shown together. In biostatistics, correlation coefficients (R) are interpreted as follows: R ≥ 0.8 indicates a very strong correlation, 0.6 ≤ R <0.8 signifies a strong correlation, 0.4 ≤ R < 0.6 represents a moderate correlation, and R < 0.4 is considered weak [34]. For example, both the dt-NSE1–3 and dt-IA123 variables exhibit strong correlations (R = 0.6–0.8) with glucose levels, while dt-IA123 shows relatively higher correlations than dt-NSE1–3.

To evaluate the blood glucose prediction performance using the Conv1D model, we analyzed a dataset comprising dt-IA123, NTh1, and NFSR data. Multicollinearity was assessed via the variance inflation factor (VIF), where thresholds of VIF > 10 indicate severe collinearity and thresholds of VIF > 5 suggest moderate concerns. For the IA123 datasets across the five subjects, the VIF values ranged from 1.0 to 2.9, confirming negligible multicollinearity. While neural networks are inherently tolerant to correlated features, extreme multicollinearity (VIF > 5) can destabilize gradient-based weight optimization or induce overfitting, ultimately degrading generalization. The observed low VIF values validate the dataset’s suitability for stable model training and reliable glucose prediction, aligning with guidelines for robust predictive analytics in physiological signal processing.

## 3. Results and Discussion

In this study, the blood glucose prediction performance was evaluated using four key metrics: root-mean-square error (RMSE), coefficient of determination (*R*^2^), mean absolute relative difference (MARD), and Clarke error grid (CEG) analyses. The RMSE was calculated as follows:

(5) $$RMSE=\sqrt{\left(1/n\right)\sum_{i=1}^{n}{\left(x_{i}-y_{i}\right)}^{2}}.$$

where *x_i_* and *y_i_* represent the reference and predicted glucose values, respectively, and *n* is the sample size. The *R*^2^ was computed as follows:

(6) $$R^{2}=1-\sum_{i=1}^{n}{\left(x_{i}-y_{i}\right)}^{2}/\sum_{i=1}^{n}{\left(x_{i}-X\right)}^{2}.$$

where *X* denotes the mean of the measured blood glucose concentrations, quantifying the proportion of variance explained by the model. The MARD was determined using

(7) $$MARD=\left(1/n\right)\sum_{i=1}^{n}100\%\cdot \left|x_{i}-y_{i}\right|/x_{i}$$

reflecting the average relative deviation between the prediction and reference values. These formulas align with established definitions in glucose monitoring research, ensuring comparability with prior studies [10,35].

For clinical accuracy, the CEG evaluates the clinical utility by categorizing paired reference-predicted glucose values into five zones (A–E). Zone A represents clinically accurate estimates, defined as ≤20% deviation for reference glucose values > 70 mg/dL or both values < 70 mg/dL, while Zone B includes non-critical deviations (>20% discrepancy without adverse clinical consequences). Performance is assessed by the proportion of values in Zones A/B, which are considered clinically acceptable. Zones C (overtreatment), D (failure to treat hypoglycemia/hyperglycemia), and E (dangerous errors with inverse treatment) indicate escalating risks of inappropriate clinical decisions. This framework bridges statistical and clinical accuracy metrics by emphasizing actionable patient outcomes over purely numerical errors [36].

### 3.1. Characteristics of NI-CGM Frame Data

**Figure 6:**
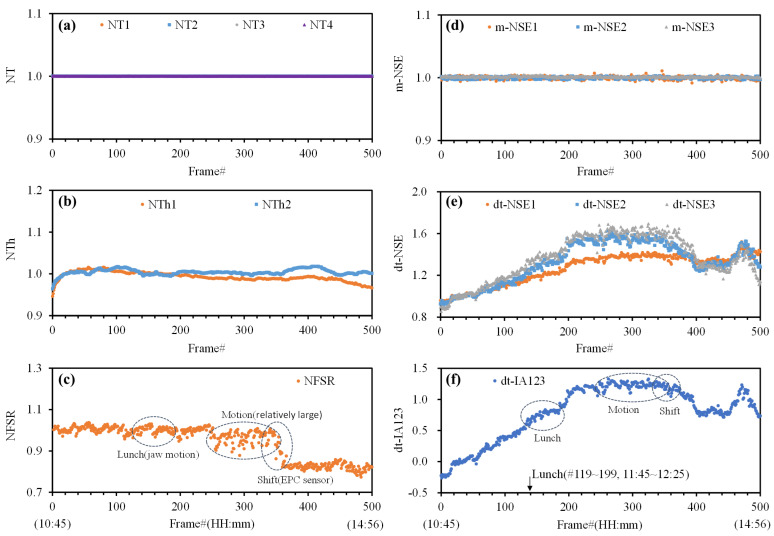
NI-CGM frame data in subject V1: (**a**) normalized temperature (T1–T4); (**b**) normalized temperature (Th1, Th2); (**c**) normalized FSR; (**d**) normalized SE of m-SE1–3; (**e**) normalized SE of dt-SE1–3; (**f**) dt-IN123, signal processed by the integral approach.

Analyzing the characteristics of NI-CGM data on a frame-by-frame basis is crucial for feature selection and dataset configuration in nonlinear neural network models. The frame size of the collected data from each subject are summarized in Table 1. In advance of the glucose prediction, we carefully examined the measured features in the frame data of the NI-CGM, as shown in Figure 6 for the example of subject V1. The Th1 temperature stabilizes approximately 10 min after the EPC sensor is worn, which is why the warmup time in this study was set to 10 min, aligning with the 20th frame. Therefore, all signals are normalized using data from the 20th frame, identified by ‘N,’ to enable effective comparisons of the signal levels. First, we analyzed the temperature monitoring signals from NT1 to NT4 for the optical sensors (LD1 to LD3, Rx1). This confirmed that the temperature of the sensor system components remained stable during continuous measurement, with an RMSE of less than 2 × 10^−4^ (Figure 6a). As a matter of course, the signals from NT1 to NT4 showed no significant correlation or uniqueness in relation to the noninvasive blood glucose predictions.

NTh1 and NTh2 denote the normalized earlobe skin temperature (Th1) and EPC sensor case temperature (Th2), respectively. Th1 reflects dynamic changes in the earlobe body temperature, while Th2 primarily indicates the ambient temperature. The variability in the earlobe skin temperature exceeded that of the ambient temperature, a trend influenced by the subject’s physiological state, the environmental conditions (e.g., lab ventilation, weather), and the sensor contact stability (Figure 6b).

The normalized FSR signal (NFSR) captures earlobe compression pressure changes induced by the EPC sensor’s spring mechanism (Figure 6c), which secures the earlobe to the Rx1. Due to the viscoelastic nature of earlobe tissue, the FSR signal exhibits high sensitivity to movement artifacts (e.g., jaw motion during speech/eating), aligning with observations in ear canal pressure sensor studies [37]. The FSR signal serves as an indicator of continuous motion artifacts during measurements, with major events (e.g., lunch intake, abrupt body motions, and EPC sensor displacement) annotated on the time-series graph. Notably, a significant FSR signal shift near frame 350 reflects the horizontal displacement of the EPC sensor from the earlobe, which introduces transient errors into concurrent optical/thermal signals while remaining within continuous artifact categories.

m-NSE1-3 represents the normalized slope efficiency derived from the ratio of power monitoring signals (mPD1–mPD3) to laser diode driving currents (LD1–LD3) during step-pulse generation. As shown in Figure 6d, m-NSE1–3 demonstrates stable step-pulse characteristics (RMSE < 2 × 10^−3^), confirming no direct correlation with blood glucose fluctuations. This stability validates the sensor system’s robustness against source power drift, a critical factor for reliable NI-CGM operation.

The normalized NSE characteristics from the Rx1, denoted as dt-NSE1 to dt-NSE3, represent the diffuse-transmission properties that reflect variability in factors such as the temperature, pressure, and blood glucose levels. While dt-NSE1–3 exhibit similar temporal trends, they differ in magnitude and display varying levels of noise across frames. These three-channel waveforms are combined into a single variable, dt-IA123, which filters out fluctuations by reducing skewness and kurtosis, as described in Section 2.3. As a result, dt-IA123 demonstrates significantly improved stability and reduced fluctuations across frames compared to the individual dt-NSE1–3 signals (Figure 6e,f).

We analyzed the temporal characteristics of multiple variables alongside NI-CGM data from subject V1, and the comparable figures for other subjects (V2–V5) were provided in the Supplementary Materials. Although behavioral events during measurements could not be fully tracked, key recorded incidents—such as jaw movement during meals, abrupt/sustained physical motions, and skin temperature fluctuations due to ventilation or ambient changes—were annotated in individual plots. These events demonstrably influenced the sensor readings, particularly through the interdependent dynamics of the NIR diffuse-transmission, temperature, and pressure parameters. The nonlinear coupling between these variables necessitated the implementation of nonlinear analytical algorithms rather than linear models.

### 3.2. Test with Individual Dataset in Five Subjects

The individual subject’s NI-CGM data were combined with their reference blood glucose data obtained using the Dexcom G7 CGM sensor for training and evaluation with the Conv1D model. The size of the NI-CGM datasets ranged from 385 to 701 frames for the five subjects, with data continuously collected over several hours to capture fluctuations in blood glucose, including during a mealtime for lunch. Each subject’s dataset was applied to the Conv1D model using an 80:20 split for training and testing, respectively. The relevant parameters and blood glucose prediction performances of the Conv1D model for each individual dataset are summarized in Table 2. Based on the analysis of the RMSE and R^2^, we observed that the prediction performance on the 20% test frames in the NI-CGM data could be affected by various factors, such as the size of the training dataset, the operational conditions of the sensor system, subject movement during testing, and variations in the earlobe thickness.

The test results demonstrate that the performance metrics, the RMSE and R^2^, are dependent on the size of the training dataset, as the Conv1D model is designed to learn local temporal patterns by moving a kernel along the time axis. For subjects V1–V3 using the EPC sensor and PS-Unit #1, increasing the training frame size improved the performance: the R^2^ rose from 0.84 to 0.95, and the RMSE dropped from 7.91 to 4.52 mg/dL (Table 2). This trend was also seen in subjects with PS-Unit #2. The minimum training data size was set at 240 frames based on the chosen hyperparameters, and all subjects (V1–V5) exceeded this threshold by using 80% of their NI-CGM data for training. Notably, subjects V1 (401 frames, 1.67 times the minimum) and V4 (561 frames, 2.24 times the minimum) achieved the best results, while subjects with fewer frames showed lower accuracy. These findings support that larger training datasets reduce the variance in neural network weights and enhance the model accuracy, especially for time-series predictions.

Subjects V4 and V5, who used the EPC sensor and PS-Unit #2, showed lower efficiency compared to subjects V1 and V2 in G1, even though their data sizes were similar or larger. In particular, the higher RMSE values for G2 indicate reduced precision between the predicted and actual values compared to V1 and V2. These performance differences may have arisen from variations between the two NI-CGM sensor systems, which employed the same MW-SP-NIRS algorithm but were configured with different hardware parameters, including different LD1–3 operation currents, Rx1 amplification gains, and optical paths.

We focused on events, activities, and earlobe characteristics during the individual subject measurements. For instance, events like jaw motion during meals or skin temperature fluctuations caused by ventilation left detectable traces in the NTh1-2 and NFSR, with corresponding changes observed in dt-IA123, albeit exhibiting a moderate impact on the glucose prediction accuracy. Subject activity levels were estimated based on posture and behavior during monitoring. For example, subject V4 remained seated with minimal movement, primarily focused on computer work, which resulted in stable sensor readings. In contrast, subject V5 engaged in frequent activities, leading to cable vibrations and motion artifacts that increased the data variability.

Variations in the earlobe thicknesses among individuals (Table 1 and Table 2) correlate with skin elasticity and directly influence the sensor contact stability. While the EPC sensor accommodates diverse earlobe thicknesses, thinner tissues reduce the contact pressure and amplify the motion sensitivity. For example, subject V5 (thinnest earlobe, 7.16% thickness reduction) exhibited pronounced data instability. The earlobe thickness showed strong correlations with the training frame size (r = 0.97) and prediction accuracy (r = 0.96) in G1. However, merging G1 and G2 diminished these correlations (r = 0.52) due to inter-system calibration discrepancies, though the earlobe thickness retained a significant association with the R^2^ (r = 0.82)

Table 2 evaluates the performance of a consolidated dataset (dt-IA123, NTh1, NFSR data) across the five subjects, demonstrating consistent glucose prediction efficacy. This feature combination was finalized after a rigorous analysis of multiple dataset configurations within the kernel–assistant–additional groups, as detailed in Section 2.5. While dt-IA123 primarily correlates with blood glucose levels, datasets excluding the NTh1 and NFSR exhibited inconsistent results: three subjects (V1, V2, V4) achieved RMSE = 5.5–9.1 mg/dL and R^2^ = 0.8–0.9, whereas two subjects (V3, V5) showed RMSE > 17.7 mg/dL and R^2^ < 0.5.

The key findings from the five-subject dataset analysis are as follows:

- Training Data Requirements: Effective temporal pattern learning requires training datasets exceeding 1.67× the minimum size threshold.
- Movement Artifacts: Subject movement during measurements reduced the accuracy, particularly in individuals with thinner earlobes, where fixed sensor pressure exacerbated the signal instability.
- Earlobe Biomechanics: Pre- and post-measurement thickness variations may stem from individual differences in skin elasticity or movement, though isolating biological versus behavioral factors requires further study.
- System Comparability: The two NI-CGM systems demonstrated comparable performances, but direct benchmarking was limited by hardware discrepancies (e.g., pressure calibration protocols, wavelength ranges).

**Figure 7:**
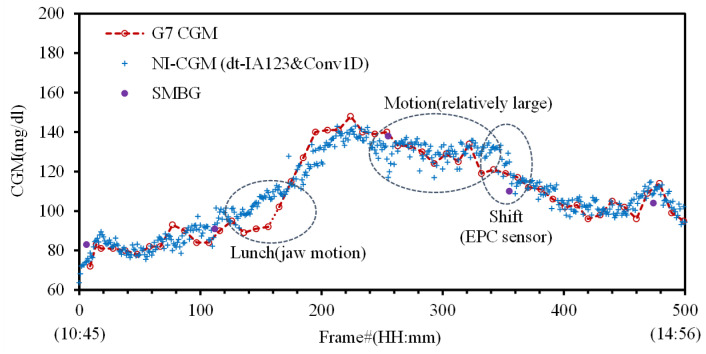
Comparison of blood glucose levels in subject V1 as measured by G7 CGM, SMBG, and NI-CGM (Conv1D model applied to consolidated dt-IA123 datasets with NTh1 and NFSR data).

In the analysis using individual subject datasets, it is valuable to compare the predicted blood glucose levels from the NI-CGM with those from the G7 CGM and SMBG. Figure 7 presents such a comparison for subject V1: NI-CGM, G7 CGM, and SMBG. The NI-CGM graph, marked with plus signs, shows the predicted blood glucose values generated by the Conv1D model using the full dataset with an 80:20 train–test split. The clinical trial procedures and data collection methods for the G7 CGM and SMBG were previously described in Section 2.4. The G7 CGM graph, indicated by open circles, displays blood glucose values recorded at 5 min intervals, with linear interpolation shown as a dotted line. The SMBG graph, represented by filled circles, presents five blood glucose values measured by finger prick at approximately 1 h intervals. In Figure 7, the MARD of the NI-CGM for subject V1 is calculated as 4.06% using Equation (7), in comparison with the G7 CGM.

### 3.3. Test with Mixed Datasets in Five Subjects

**Figure 8:**
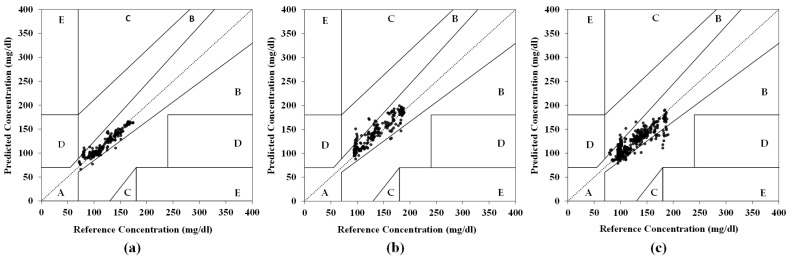
Clarke error grid (CEG) analysis of Conv1D model predictions on mixed datasets for (**a**) G1; (**b**) G2; (**c**) combined G1 and G2.

We evaluated the performance of the Conv1D model using mixed datasets from all subjects, grouped as G1 (1355 frames, three subjects) and G2 (1167 frames, two subjects), for a total of 2522 frames (see Table 3). The model training and testing were conducted with an 80:20 split using previously optimized hyperparameters. Figure 8 presents the Clarke error grid (CEG) analysis comparing the predicted blood glucose values for each group and for all subjects combined, based on the 20% test set. Table 3 summarizes the Conv1D model’s prediction accuracy (CEG Zone-A, RMSE, MARD) across groups.

The Conv1D model demonstrated a strong performance:

- G1 (EPC sensor and PS-Unit #1): The model achieved 97.0% CEG Zone-A accuracy (Figure 8a), a 5.2% MARD, and a test RMSE of 7.95 mg/dL (vs. the training RMSE: 6.77 mg/dL), indicating no overfitting.
- G2 (EPC sensor and PS-Unit #2): The model achieved 93.2% CEG Zone-A accuracy (Figure 8b), with an RMSE of 14.37 mg/dL and a 7.56% MARD.
- Combined Group (G1 + G2): The model achieved 90.9% CEG Zone-A accuracy (Figure 8c), with an RMSE of 14.13 mg/dL and an 8.44% MARD, showing slightly reduced accuracy compared to the individual-group analyses.

The CEG Zone-A and MARD show consistent trends by quantifying the relative error and clinical validity, while the RMSE exhibits higher variability due to sensitivity to absolute errors and outliers (e.g., subject V5 in Table 2, G2 in Table 3), particularly under unstable conditions such as exercise-induced temperature/pressure fluctuations. For the NI-CGM validation, the MARD + CEG assesses the clinical applicability, whereas the RMSE identifies system-specific limits like the maximum tolerable glucose spikes (≥200 mg/dL/min), critical for hypoglycemia/hyperglycemia risk management.

The mixed-dataset evaluations confirmed that both NI-CGM systems (EPC sensor and PS-Units #1 and #2) deliver clinically acceptable performances—with a CEG Zone-A ratio above 90% (Zone-A+B: 100%) and a MARD below 8.5—when using the same MW-SE-NIRS algorithm, despite hardware variations in the LD current and optical coupling. This highlights the effectiveness of our NI-CGM system and MW-SE-NIRS algorithm, which integrate multi-wavelength diffuse-transmission NIR, temperature, and pressure signals from the earlobe.

### 3.4. Cross-Subject Generalization Test

To evaluate the generalization capability of the Conv1D model, we conducted cross-subject tests by separating the training and testing datasets according to individual subjects. As summarized in Table 4, the model showed a marked decline in performance when evaluated on subjects whose data were not included in the training set. For example, in the intra-group tests (G1: V1–V3), training on V2 and V3 and testing on V1 achieved moderate results, with an RMSE of 25.06 mg/dL and a MARD of 16.54% (within the FDA’s 15–20% acceptable error margin). In contrast, training on V1 and V2 and testing on V3 resulted in a statistically significant performance drop, with the RMSE increasing to 58.66 mg/dL and the MARD to 62.57%, highlighting the impact of subject-specific variability.

In the cross-group tests, training on V1, V2, and V3 and testing on V4 resulted in an RMSE of 69.07 mg/dL and a MARD of 30.21%. Expanding the training set to include V5 (training on V1, V2, V3, and V5, testing on V4) yielded partial improvements, with the RMSE decreasing to 50.50 mg/dL and the MARD to 24.98%. However, when training on V1, V2, V3, and V4 and testing on V5, the performance slightly decreased compared to training on V1, V2, and V3 and testing on V5, as detailed in Table 4. These findings highlight the considerable challenge of accurately predicting blood glucose levels for new subjects in the absence of their data in the training set. This underscores the importance of personalized calibration or hybrid modeling approaches, supported by large and diverse clinical datasets, to improve generalizability [34,35,36,37,38].

Recent research demonstrates that accurately predicting blood glucose levels for new subjects without their feature data in the training set is challenging, highlighting the importance of personalized calibration and hybrid modeling approaches, particularly when supported by large, diverse clinical datasets. Studies show that inter-subject variability—such as physiological differences and lifestyle factors—significantly limits the generalizability of standard machine learning models for glucose prediction [38,39]. Although advanced models like LSTM and self-attention networks generalize better than simpler models, their accuracy still declines for unseen individuals or domains unless the training data are sufficiently comprehensive. To address these challenges, strategies such as personalized calibration [40], hybrid and domain generalization models [41,42], and the use of large-scale clinical data have been proposed.

In this study, we explicitly acknowledge the small sample size (n = 5) as a limitation that restricts the generalizability of our findings. To address this, we plan to conduct larger-scale clinical trials in future work to further validate our approach. Ultimately, overcoming the challenge of predicting blood glucose for new subjects requires both advanced modeling techniques and access to extensive, heterogeneous clinical datasets—an essential foundation for developing reliable and clinically useful noninvasive glucose monitoring systems.

## 4. Conclusions

This study demonstrates the feasibility of noninvasive continuous glucose monitoring (NI-CGM) using the earlobe, which integrates an EPC sensor, a PS-Unit for temperature/pressure measurement, and the novel MW-SE-NIRS algorithm. In a clinical trial with five participants using two NI-CGM systems, the system achieved 90.9% CEG Zone-A accuracy, a 14.13 mg/dL RMSE, and an 8.44% MARD within a reference glucose range of 78–191 mg/dL (validated against the Dexcom G7 CGM, MARD~9%). Notably, in three participants using the same NI-CGM system (EPC sensor and PS-Unit #1), the performance improved to 97.0% CEG Zone-A accuracy and a 5.2% MARD, highlighting enhanced reliability. These results suggest that earlobe-based MW-SE-NIRS offers advantages over traditional finger-based methods. The findings underscore the importance of personalized data modeling and sensor optimization to improve accuracy. The Conv1D model effectively captures short-term glucose dynamics and processes multidimensional data, supporting scalability. Future studies should prioritize hybrid modeling approaches and large-scale clinical validation to enhance the generalizability across diverse populations.

## Figures and Tables

**Table 1:** Volunteer, earlobe thickness, and NI-CGM sensor summary.

Volunteer		Earlobe Thickness (mm)			NI-CGM Sensor
Subject	HbA1c	Before	After	Variation	Prototype
V1	Normal	4.06	4.09	+0.74%	EPC and PS-Unit #1
V2	Normal	4.80	4.72	−1.67%	
V3	Pre-diabetic	4.47	4.08	−8.72%	
V4	Pre-diabetic	3.68	3.67	−0.27%	EPC and PS-Unit #2
V5	Normal	3.77	3.50	−7.16%	

**Table 2:** Test results using individual datasets from five subjects.

Volunteer			Earlobe Thickness	NI-CGM Data		Performances	
Group	Subject	Activity	Variation	System#	Frame#	RMSE	R^2^
G1	V1	Low	0.74%	EPC and PS-Unit #1	501	4.52	0.95
	V2	Middle	–1.67%		469	5.13	0.92
	V3	High	–8.72%		385	7.91	0.84
G2	V4	Low	–0.27%	EPC and PS-Unit #2	701	7.85	0.95
	V5	High	–7.16%		466	14.90	0.75

**Table 3:** Performance of the Conv1D model using mixed datasets from G1 and/or G2.

Volunteers		Frame#		Test Results		
Group	Subjects	Mixed	Test	CEG Zone-A	RMSE	MARD
G1	V1~V3	1355	271	97.0%	7.95	5.20%
G2	V4~V5	1167	234	93.2%	14.37	7.56%
G1 and G2	V1~V5	2522	505	90.9%	14.13	8.44%

**Table 4:** Cross-subject test results evaluating generalization capability.

Classification	Cross-Subjects		Test Results	
	Training	Testing	RMSE	MARD
Intra-Group (G1)	V2 + V3	V1	25.06	16.54
	V1 + V3	V2	39.73	24.95
	V1 + V2	V3	58.66	62.57
Cross-Group (G1, G2)	V1 + V2 + V3	V4	69.07	30.21
	V1 + V2 + V3 + V5	V4	50.50	24.98
	V1 + V2 + V3	V5	25.08	17.33
	V1 + V2 + V3 + V4	V5	28.76	20.91

## Data Availability

The original contributions presented in this study are included in the article and its Supplementary Materials. Further inquiries can be directed to the corresponding author with clear reasons.

## Supplementary Materials

The following supporting information can be downloaded at: https://www.mdpi.com/article/10.3390/bios15070406/s1. Supplementary figures comparing the other subjects (V2–V5) in Figure 6.

## Abbreviations

The following abbreviations are used in this manuscript:

NI-CGM	Noninvasive Continuous Glucose Monitoring
MW-SE-NIRS	Multi-Wavelength Slope Efficiency Near-Infrared Spectroscopy
SE	Slope Efficiency
EPC	Earlobe-Parallel Clip
PS-Unit	Portable Sensor Unit
FSR	Force-Sensitive Resistor (pressure sensor signal)
LD	Laser Diode (light source)
L1–3	Laser Output Signals From LD1–3 (wavelengths λ_1_, λ_2_, λ_3_)
mPD1-3	Photodiodes, Monitoring Output Intensities From LD1–LD3.
Rx1	Receiver 1 (optical receiving signal)
Th1	Thermistor 1 (earlobe skin temperature)
Th2	Thermistor 2 (sensor case/ambient temperature)
T1–T3, T4	Temperatures of LD1-LD3 and Rx1
NT1–4	Normalized T1-4
NTh1–2	Normalized Th1-2
NSE	Normalized Slope Efficiency
m-NSE1–3	Generated Power Monitoring NSE From mPD1–3, as defined in Equation (2)
dt-NSE1–3	Diffused Transmission NSE From Rx1, as defined in Equation (3)
dt-IA123	Integrated Value of dt-NSE1–3, as defined in Equation (4)
Conv1D	1D Convolutional Neural Network
CEG	Clarke Error Grid
MARD	Mean Absolute Relative Difference
RMSE	Root Mean Square Error
