# Supplementary material for: Noninvasive Continuous Glucose Monitoring Using Multimodal Near-Infrared, Temperature, and Pressure Signals on the Earlobe
Source: Biosensors (Basel). 2025 Jun 24;15(7):406. doi: 10.3390/bios15070406 (PMC12292979; doi:10.3390/bios15070406)
Supplement: Supplementary file 1 [file biosensors-15-00406-s001.zip › biosensors-3661810-supplementary.pdf]

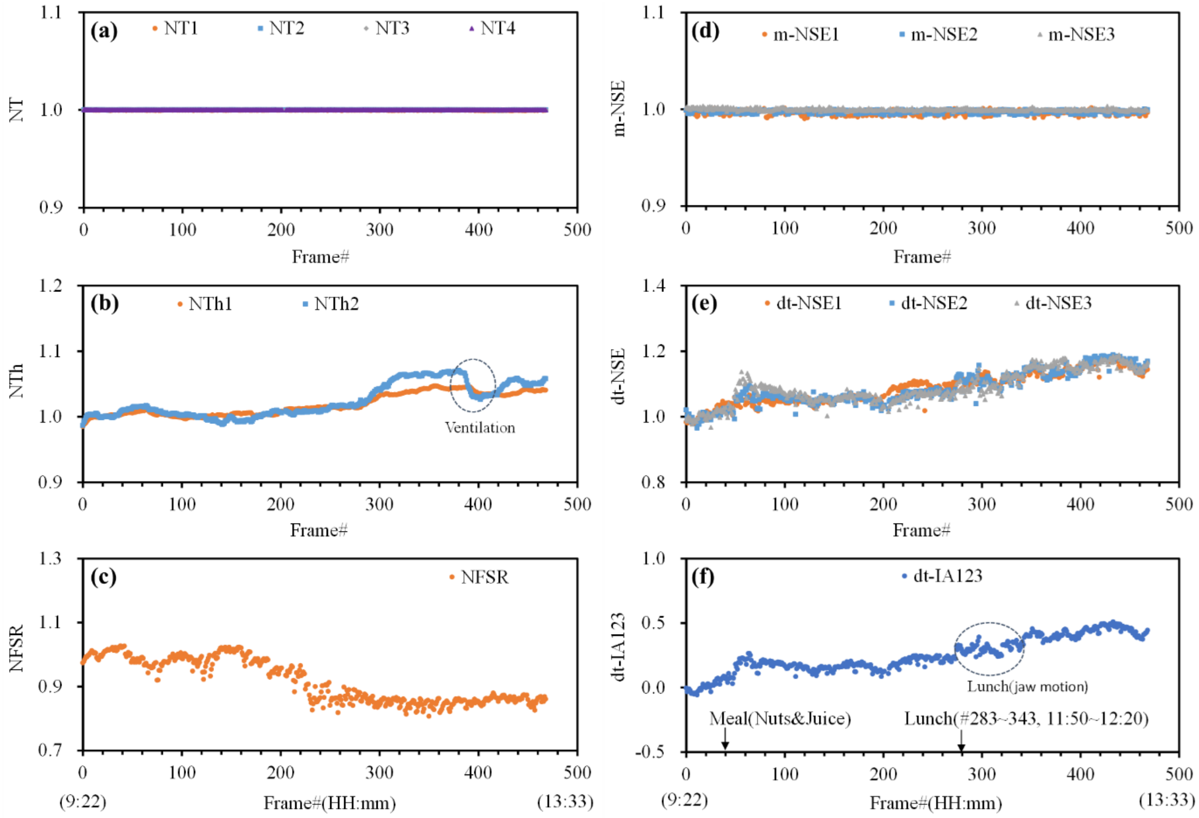

Figure 6(V2). NI-CGM frame data in subject V2: (a) Normalized temperature (T1–T4); (b) Normalized temperature (Th1, Th2); (c) Normalized FSR; (d) Normalized SE of m-SE1–3; (e) Normalized SE of dt-SE1–3; (f) dt-IN123, signal processed by the integral approach.

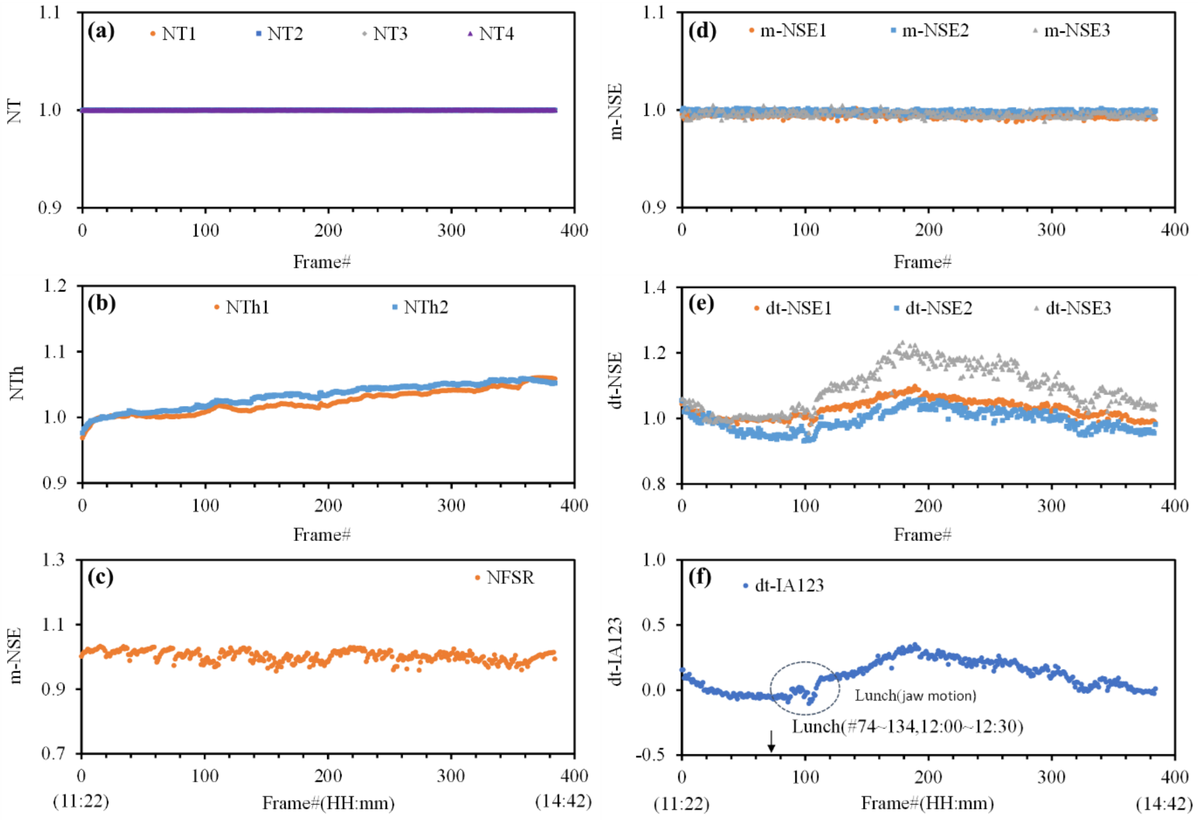

Figure 6(V3). NI-CGM frame data in subject V3: (a) Normalized temperature (T1–T4); (b) Normalized temperature (Th1, Th2); (c) Normalized FSR; (d) Normalized SE of m-SE1–3; (e) Normalized SE of dt-SE1–3; (f) dt-IN123, signal processed by the integral approach.

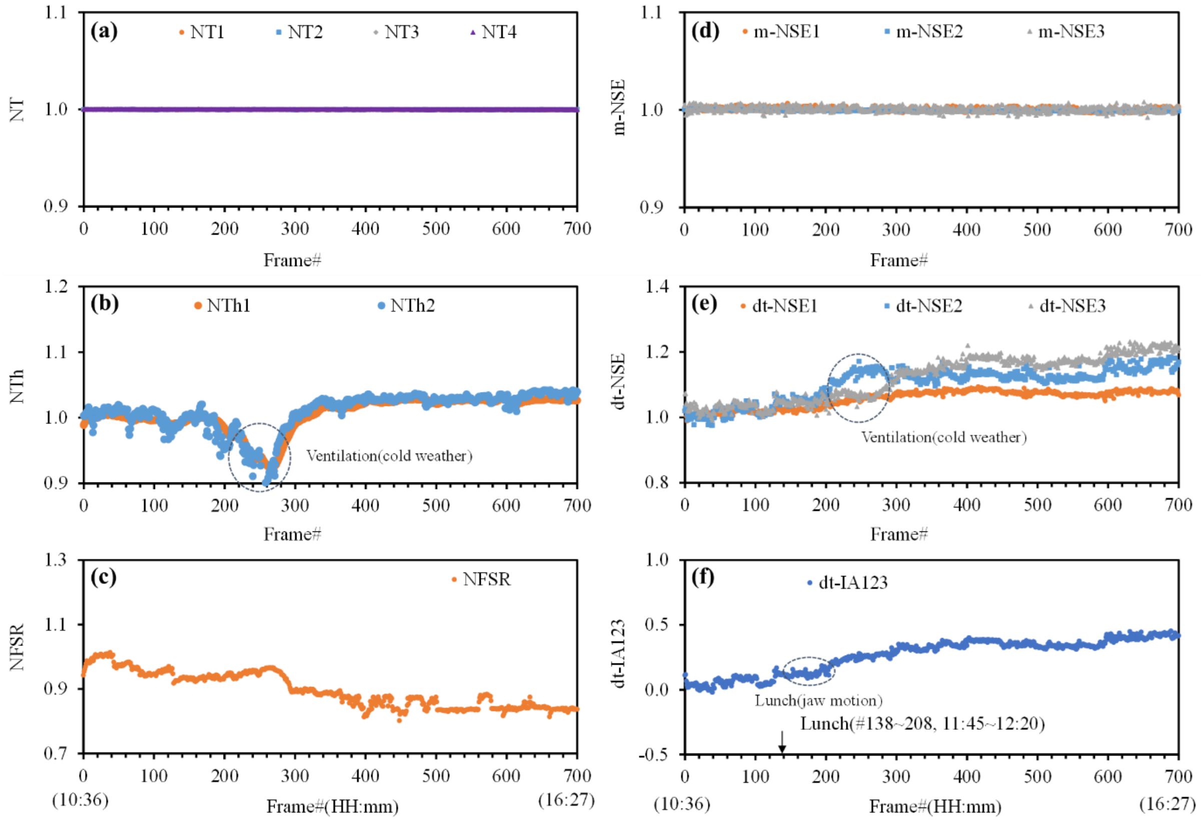

Figure 6(V4). NI-CGM frame data in subject V4: (a) Normalized temperature (T1–T4); (b) Normalized temperature (Th1, Th2); (c) Normalized FSR; (d) Normalized SE of m-SE1–3; (e) Normalized SE of dt-SE1–3; (f) dt-IN123, signal processed by the integral approach.

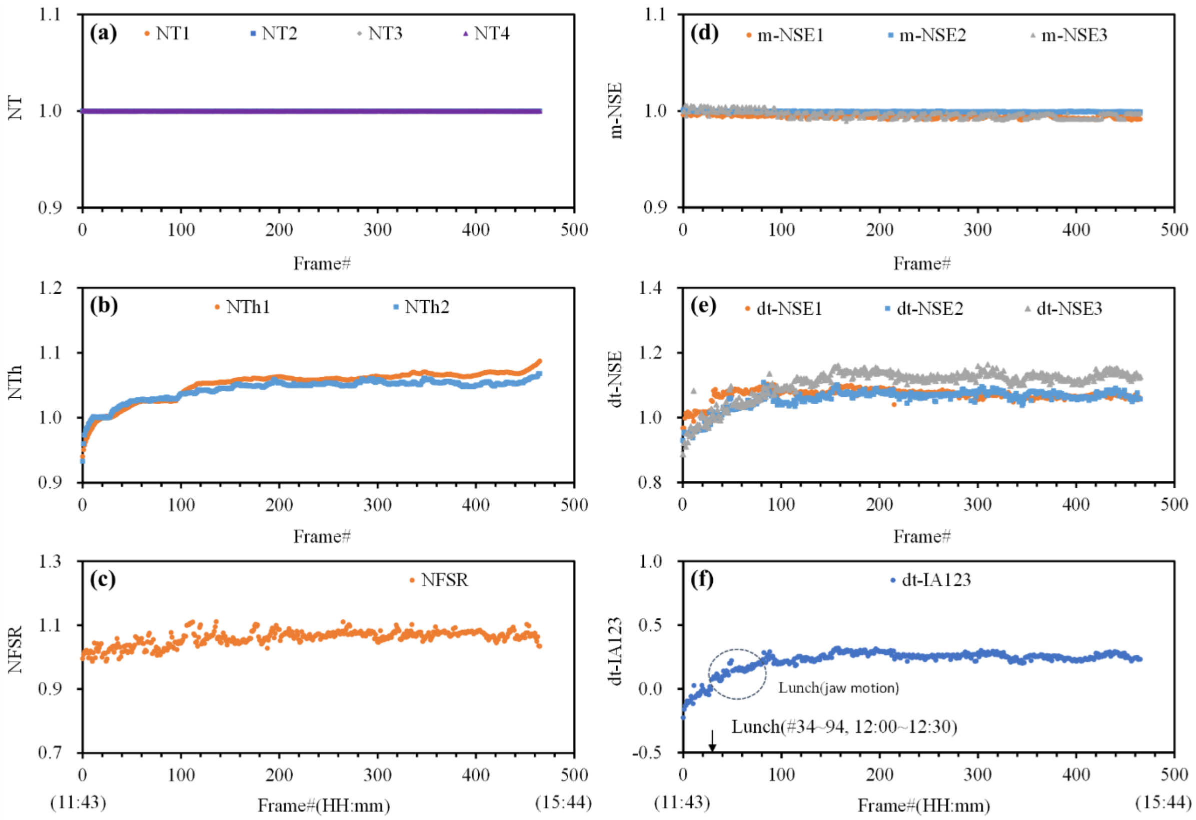

Figure 6(V5). NI-CGM frame data in subject V5: (a) Normalized temperature (T1–T4); (b) Normalized temperature (Th1, Th2); (c) Normalized FSR; (d) Normalized SE of m-SE1–3; (e) Normalized SE of dt-SE1–3; (f) dt-IN123, signal processed by the integral approach.
